# Development and Optimization of Beeswax–Coriander Essential Oil-Based Nanostructured Lipid Carriers for Encapsulation of Anthocyanin-Rich Barberry Extract

**DOI:** 10.3390/foods15101685

**Published:** 2026-05-12

**Authors:** Sima Khezri, Babak Ghanbarzadeh, Hamed Hamishehkar, Maryam Mohammadi, Ali Ehsani, Pasquale M. Falcone

**Affiliations:** 1 Student Research Committee, Tabriz University of Medical Sciences, Tabriz 5165665931, Iran; simakhezri92@gmail.com; 2 Department of Food Science and Technology, Faculty of Nutrition and Food Sciences, Tabriz University of Medical Sciences, Tabriz 5166614711, Iran; 3 Department of Food Science and Technology, Faculty of Agriculture, University of Tabriz, Tabriz 5166616471, Iran; ghanbarzadeh@tabrizu.ac.ir; 4 Department of Food Engineering, Faculty of Engineering, Near East University, 99138 Mersin, Turkey; 5 Drug Applied Research Center, Tabriz University of Medical Sciences, Tabriz 5165665811, Iran; hamishehkar.hamed@gmail.com; 6 New Material and Green Chemistry Research Center, Khazar University, 41 Mehseti Street, Baku AZ1096, Azerbaijan; 7 Department of Food Science and Engineering, Faculty of Agriculture, University of Kurdistan, Sanandaj 6617715175, Iran; ma.mohammadi@uok.ac.ir; 8 Department of Agricultural, Food and Environmental Sciences, University Polytechnical of Marche, Brecce Bianche 10, 60131 Ancona, Italy; pm.falcone@univpm.it

**Keywords:** *Berberis vulgaris*, anthocyanins, lipid nanoparticles, double emulsion, stability, surfactants, antioxidant activity

## Abstract

Nanostructured lipid carriers (NLCs) are colloidal delivery systems developed to address the low stability and limited bioavailability of sensitive active compounds. In this study, anthocyanin-rich barberry extract-loaded NLCs were prepared by a water-in-oil-in-water double emulsion method, using beeswax as the solid lipid and coriander essential oil as the liquid lipid. A combined D-optimal mixture design was employed to evaluate the effect of surfactant ratios (Tween 80/Tween 20 and polyglycerol ester (PGE)/polyglycerol polyricinoleate (PGPR)) on particle size, polydispersity index (PDI), zeta potential, and encapsulation efficiency. The optimized formulation suggested by Design-Expert^®^ software was obtained at 90/10 Tween 80/Tween 20 and 90/10 PGE/PGPR ratios and showed a particle size of 94.25 nm, PDI of 0.18, zeta potential of −23.4 mV, and encapsulation efficiency of 74%. The experimental values were in close agreement with the predicted responses. TEM observations indicated spherical morphology at the nanoscale, while FTIR, DSC, and XRD analyses confirmed successful incorporation of barberry extract into the lipid matrix and a less ordered crystalline structure. During one month of storage, the optimized NLC was more stable at 4 °C compared with 25 °C and showed higher antioxidant activity than the free extract. It also exhibited a higher inhibitory effect against *S. aureus* and *E. coli* than the free form in MIC and MBC assays. Overall, the developed NLCs could serve as an effective carrier system to improve the stability of anthocyanin-rich barberry extract and extend its application in food formulations.

## 1. Introduction

Barberry (*Berberis vulgaris* L.), a shrub from the Berberidaceae family, is widely distributed across Europe and Asia. Seedless barberry is consumed in traditional foods and medicine and has also been used in the production of juice, syrup, and dried products [1]. Studies have indicated that barberry fruit possesses antioxidant, antimicrobial, and anticancer activities and has beneficial effects on the vascular and nervous systems [2]. These biological properties are associated with its phenolic composition, among which anthocyanins are the most prominent compounds contributing to both the red color of the fruit and its health-promoting effects [3]. Due to their high compatibility with biological systems, water solubility, and nontoxic nature, anthocyanins have received considerable attention as natural colorants in food products [4]. However, their application in food systems is still limited by their low stability during processing, storage, and distribution, as well as their poor bioavailability caused by rapid metabolism and low absorption [5]. This limitation is mainly attributed to the unstable flavylium cation core of anthocyanins, which makes them highly vulnerable to chemical and biochemical degradation. Their instability is strongly influenced by adverse environmental factors such as pH, temperature, light, oxygen, enzymes, and other reactive compounds including ascorbic acid [6].

To address these problems, various encapsulation systems have been explored to improve the stability and bioavailability of anthocyanins. However, several limitations have been reported in these systems. Spray-drying, although widely used due to its industrial scalability, involves elevated temperatures that may accelerate anthocyanin degradation. In addition, the size and morphology of the resulting particles are not well controlled. Liposomal systems often exhibit limited physical stability during storage and are prone to oxidative degradation [2,6]. Solid lipid nanoparticles (SLNs) may suffer from low loading capacity and expulsion of encapsulated compounds during polymorphic transitions [7]. To overcome these drawbacks, nanostructured lipid carriers (NLCs) as one type of lipid-based delivery systems, offer additional advantages including higher efficiency and loading capacity, improved physicochemical stability, enhanced bioavailability (2- to 5-fold) and controlled release of bioactive compounds. These carriers are characterized by a lipid core composed of a mixture of solid and liquid lipids, which is stabilized in an aqueous phase by surfactants [8,9]. However, since the lipid matrix of NLC is hydrophobic, highly hydrophilic compounds such as anthocyanins are expected to be poorly encapsulated because of their strong tendency to partition into the outer aqueous phase during preparation [10]. Among the various methods developed for the production of lipid nanoparticles, the water-in-oil-in-water (W/O/W) double emulsion technique has been recommended for the encapsulation of hydrophilic compounds [11]. In this method, the target compounds are first dissolved in an internal aqueous phase (W_1_) and emulsified into melted lipids to form a primary W_1_/O emulsion, which is then re-emulsified into a second aqueous surfactant phase (W_2_) to obtain a W_1_/O/W_2_ double emulsion. Upon cooling, the lipid matrix recrystallizes to form NLCs [12]. Beeswax, a natural solid lipid, has been employed as a matrix substance for the preparation of stable lipid nanoparticles with favorable loading and release characteristics. Beeswax mainly contains esters of long-chain aliphatic alcohols (C30–C32) and fatty acids (C16–C18), including palmitate, palmitoleate, hydroxypalmitate, and oleate. It is considered food-grade, low-cost, non-toxic, and widely available. Other advantages include a tunable microstructure, resistance to environmental conditions such as pH and salt, and structural stability during the homogenization process. In addition, its orthorhombic crystal structure and low polymorphic transition rate contribute to enhanced physical stability in solid lipid-based systems [13,14].

Coriander essential oil was considered the liquid lipid phase of the formulation in this study. Coriander (*Coriandrum sativum* L.) is a widely cultivated medicinal and aromatic plant from the Apiaceae family, known for its nutritional value and therapeutic applications. Its seeds contain up to 1% essential oil, with linalool being the predominant component (57–72%). The oil also includes other terpenes and oxygenated compounds such as α-pinene, γ-terpinene, camphor, limonene, geranyl acetate, and myrcene. Coriander essential oil has been shown to have a broad spectrum of antimicrobial activity against Gram-positive and Gram-negative bacteria, yeasts, and molds, as well as antioxidant, antidiabetic, anticancer, and antimutagenic effects [15,16].

To achieve a stable and functional double emulsion-based nanocarrier, careful selection and optimization of surfactants are essential. In W/O/W systems, both hydrophilic and lipophilic emulsifiers are required to stabilize the internal and external interfaces. Polyglycerol esters (PGEs) are nonionic surfactants with a wide hydrophilic-lipophilic balance (HLB) range (3–14), depending on the type and number of fatty acids and glycerol units in their structure. Triglycerol monostearate (TGMS), a type of PGE with an HLB of ~5–7, was used as a lipophilic emulsifier to stabilize the W_1_/O interface. Polyglycerol polyricinoleate (PGPR), also a PGE with an HLB of ~2–3, served as a strong emulsifier in W/O systems. Tween 20 and Tween 80, with HLB values of 16.7 and ~15 respectively, were selected for their high interfacial activity and frequent use in food-grade formulations. These four emulsifiers were chosen based on their functional roles, biocompatibility, and GRAS status. The present study aimed to develop and optimize a food-grade W_1_/O/W_2_ double emulsion-based nanostructured lipid carrier composed of beeswax and coriander essential oil for the encapsulation of anthocyanin-rich barberry extract. The effect of surfactant ratios on particle size, polydispersity index (PDI), zeta potential (ZP), and entrapment efficiency (EE) was investigated using Design of Experiments (DOE). The optimized formulation was further evaluated for morphological characteristics, antioxidant activity, antimicrobial properties, and storage stability. The findings of this study may lead to the development of more effective carrier systems for water-soluble bioactive compounds.

## 2. Materials and Methods

### 2.1. Materials

Dried seedless barberries (*Berberis vulgaris* L.) were obtained from a local market in Qaen (South Khorasan Province, Iran) and stored at −18 °C until use. Coriander (*Coriandrum sativum* L.) essential oil was supplied by Zardband Pharmaceuticals Co. (Tehran, Iran), and beeswax was purchased from Yas Sepidvash Co. (Tehran, Iran). Tween 80, Tween 20, polyglycerol ester (PGE), polyglycerol polyricinoleate (PGPR), dimethyl sulfoxide (DMSO, ≥99.9%), ethanol (96% *v*/*v*), sodium carbonate, sodium acetate, potassium chloride, acetic acid, hydrochloric acid, and microbial culture media were obtained from Merck Chemical Co. (Darmstadt, Germany). 2,2-diphenyl-1-picrylhydrazyl (DPPH, ≥95%), and citric acid (≥99.5%) were acquired from Sigma-Aldrich Co. (St. Louis, MO, USA). Microbial strains were provided by the Pasteur Institute of Iran (Tehran, Iran). All chemicals used in this study were of analytical grade.

### 2.2. Preparation of Anthocyanin-Rich Extract from Barberry

The extraction procedure was carried out following the method described by Jaberi et al. [17], with some modifications. Dried seedless barberries were first ground using an electric blender, and then a hydroalcoholic solvent composed of ethanol and distilled water (80:20 *v*/*v*), acidified with 2% citric acid, was added at a solid-to-solvent ratio of 1:20 (*w*/*v*). The mixture was gently stirred with a magnetic stirrer for 24 h at room temperature (25 ± 2 °C) in the dark. After extraction, the supernatant was vacuum-filtered through Whatman No. 1 filter paper to remove coarse particles, followed by centrifugation (Universal 320, Hettich, Tuttlingen, Germany) at 5000 rpm for 10 min. The solvent was subsequently evaporated using a rotary evaporator (Laborota 4002, Heidolph, Schwabach, Germany) at 45 °C under reduced pressure. The resulting concentrated extract was then lyophilized (ALPHA 1–4 LD freeze dryer, Martin Christ, Osterode am Harz, Germany) at −30 °C and 0.07–0.1 mbar for 2 days, and stored at −18 °C in the dark until further use.

### 2.3. Total Anthocyanin Concentration of Barberry Extract

The total anthocyanin content (TAC) of the barberry extract (BE) was determined using the pH-differential spectrophotometric method, which is based on the pH-dependent structural transformation of anthocyanins in two buffer systems adjusted to pH 1.0 and 4.5. For this purpose, the extract was separately diluted with potassium chloride buffer (0.025 M, pH 1.0) and sodium acetate buffer (0.4 M, pH 4.5), and the resulting solutions were kept at room temperature for 15 min. The absorbance was then measured at 520 and 700 nm using a UV–visible spectrophotometer with distilled water as the blank. The results were expressed as milligrams of cyanidin-3-glucoside equivalents per gram of dry barberry extract, according to the following Equations (1) and (2):

Δ*A* = (*A*_520_ − *A*_700_) _pH 1.0_ − (*A*_520_ − *A*_700_) _pH 4.5_ (1)

(2) $$TAC=\frac{\Delta A\times MW\times DF\times 100}{\varepsilon \times L\times Wt}$$

where Δ*A* is the absorbance difference; MW is the average molecular weight of cyanidin-3-glucoside (449.2 g mol^−1^); DF is the dilution factor; ε is the molar extinction coefficient for cyanidin-3-glucoside (26,900 L mol^−1^ cm^−1^); L is the path length (1 cm) and Wt is the sample weight (g) [18].

### 2.4. Preparation of NLCs

A high-shear homogenization–ultrasonication technique was employed to prepare a W/O/W double emulsion system for the fabrication of NLCs containing anthocyanin-rich barberry extract. To prepare the inner aqueous phase (W_1_), barberry extract was dissolved in 1 mL of twice-distilled water using a magnetic stirrer. The lipid phase was composed of 350 mg of beeswax as the solid lipid and 150 mg of coriander essential oil as the liquid lipid (70:30 *w*/*w*). This ratio was selected based on previous studies and preliminary formulation trials [13,14,19]. The lipid phase was then melted in the water bath at 70 °C (approximately 5 °C above the melting point of beeswax). Then, 250 mg of PGE and PGPR (in different proportions, according to Table 1) were added to this lipid phase as lipophilic surfactants. The inner aqueous phase (W_1_) was slowly added to the melted lipid phase using a micropipette under high-shear homogenization (Heidolph, Kelheim, Germany) at 5000 rpm to produce the W_1_/O primary emulsion. In the next step, the external aqueous phase (W_2_) was prepared by dissolving Tween 20 and Tween 80 (1% *w*/*v*) in 25 mL of distilled water and preheated to match the lipid phase temperature. To form the double emulsion (W_1_/O/W_2_), the aqueous phase was added to the previously prepared W_1_/O emulsion under high shear in a drop-by-drop manner. The resulting emulsion was further stirred at 20,000 rpm for 20 min, followed by sonication using a probe sonicator (UP200H, Hielscher, Teltow, Germany) for 10 cycles of 1 min ultrasonication (0.5 cycles/s, 80% amplitude, 200 W, 24 kHz), with 1 min intervals between cycles [20]. Subsequently, the final emulsion was stored at −18 °C for 10 min for crystallization and forming NLCs. Final NLC solutions were kept at refrigerator temperature (4 °C) until experiments.

### 2.5. Physicochemical Characteristics of NLCs

#### 2.5.1. Measurement of Particle Size, Polydispersity Index, and Zeta Potential

The particle size, polydispersity index (PDI), and zeta potential of the prepared nanoparticles were evaluated using a Zetasizer (Nano ZS ZEN 3600, Malvern Instrument, Malvern, UK). This instrument utilizes dynamic light scattering (DLS) to quantify the intensity of scattered light caused by particle motion. Prior to analysis, samples were diluted 1:20 with deionized water to avoid multiple scattering effects. The Z-average, reported as the average hydrodynamic diameter, was used to represent particle size. PDI values, ranging from 0 to 1, characterize the homogeneity of dispersion, with values greater than 0.3 indicating significant heterogeneity. Zeta potential (electrical charge) was measured by electrophoretic light scattering (ELS) using the same instrument, which calculates the zeta potential based on the electrophoretic mobility of the particles via the Helmholtz–Smoluchowski equation. The zeta potential reflects the surface charge of nanoparticles and is considered an important indicator of colloidal stability. All measurements were typically carried out at room temperature (22–25 °C) and performed in triplicate to ensure accuracy [21].

#### 2.5.2. Transmission Electron Microscopy

The morphology and surface characteristics of the optimized NLC formulation were studied using transmission electron microscopy (TEM). A drop of the NLC dispersion (diluted 1:10 *v*/*v* with deionized water) was placed onto a carbon-coated copper grid and negatively stained with 2% uranyl acetate for 2 min at room temperature. Excess stain was gently blotted off with filter paper, and the grids were left to air-dry before observation by TEM (LEO 906, Zeiss, Oberkochen, Germany) [22].

#### 2.5.3. Entrapment Efficiency and Entrapment Stability

The Entrapment efficiency (EE%) of the prepared NLCs was calculated by determining the amount of free anthocyanins in the dispersion medium [20]. A predetermined amount of the sample was diluted with distilled water (1:10), transferred into Amicon^®^ Ultra-15 centrifugal filter units (30 kDa, Millipore, Burlington, MA, USA), and centrifuged at 4000 rpm for 10 min at 25 °C to separate the unencapsulated fraction. The centrifugation conditions were selected to minimize possible disruption of the NLC structure. The obtained filtrate was then analyzed for anthocyanin content using UV–vis spectrophotometry, as described in Section 2.3. The total amount of anthocyanins was regarded as the sum of encapsulated and free contents, and EE% was calculated using the following Equation (3):

(3) $$EE\left(\%\right)=\frac{\mathrm{Total}\;\mathrm{amount}\;\mathrm{of}\;\mathrm{anthocyanins}-\mathrm{Free}\;\mathrm{anthocyanins}}{\mathrm{Total}\;\mathrm{amount}\;\mathrm{of}\;\mathrm{anthocyanins}}\times 100$$

The stability of the optimized NLCs was evaluated under dark conditions at 4 and 25 °C for one month. The samples were stored in polyethylene microtubes, and the loaded amount retention (%) of anthocyanins in NLCs was determined on days 1, 7, 14, 21, and 28 to investigate the effect of storage time and temperature according to Equation (4):

(4) $$\mathrm{Retention}\left(\%\right)=\frac{\mathrm{Entrapped}\;\mathrm{anthocyanins}\;\mathrm{on}\;\mathrm{day}\;t}{\mathrm{Entrapped}\;\mathrm{anthocyanins}\;\mathrm{on}\;\mathrm{day}\;1}\times 100$$

Retention (%) was normalized to the value measured at day 1, which was set as 100% and used as the reference point for all subsequent time points. The day-0 value corresponds to the initial stage immediately after preparation and was not used for normalization.

#### 2.5.4. Fourier Transform-Infrared Spectroscopy (FTIR)

FT-IR spectra of lyophilized optimized BE-loaded NLC, blank NLC, and pure barberry extract were recorded using an FTIR spectrometer (Tensor 27, Bruker, Ettlingen, Germany) to evaluate possible interactions between the components. The samples were thoroughly mixed with KBr at a 1:10 ratio to form pellets. Spectra were collected over the range of 400–4000 cm^−1^ with a resolution of 4 cm^−1^ [23].

#### 2.5.5. Differential Scanning Calorimetry (DSC)

The thermal behaviour of lyophilized optimized BE-loaded NLC, blank NLC, and pure BE was evaluated using a differential scanning calorimeter (DSC; model 200 F3 Maia, NETZSCH, Selb, Germany). About 5 mg of each sample was weighed into standard aluminum pans and sealed. The samples were heated from 0 °C to 250 °C at a scan rate of 10 °C/min. An empty aluminum pan was used as a reference. Melting and transition temperatures were determined from the resulting thermograms as the peak values of the corresponding endothermic transitions [24].

#### 2.5.6. X-Ray Diffraction Analysis

X-ray diffraction (XRD) analysis was performed to study the crystallinity and polymorphic transitions of the lyophilized optimized BE-loaded NLC, blank NLC, and pure BE. Scanning was carried out using an X-ray diffractometer (Tongda TD-3700, Dandong, China) at 40 kV and 30 mA with Cu Kα radiation (λ = 1.5406 Å). The diffraction patterns were collected over a 2θ range of 10° to 70° with a scanning rate of 0.04°/min at room temperature (25 ± 2 °C) [7].

In these analyses, lyophilized samples were used for FTIR, DSC, and XRD to evaluate the solid-state properties of the system. The results reflect the characteristics of the dried lipid matrix rather than the original W/O/W emulsion structure.

#### 2.5.7. Antioxidant Properties

The DPPH (2,2-diphenyl-1-picrylhydrazyl) free radical scavenging technique was used to determine the antioxidant activity of the samples [25]. Briefly, 100 µL of the NLC samples was mixed with 900 µL of 96% ethanol and incubated at 60 °C for 15 min. The mixture was then centrifuged at 12,000 rpm for 10 min to separate the supernatant. This ethanol-assisted extraction step was used to disrupt the NLC structure and release the encapsulated bioactive compounds prior to analysis. Subsequently, 1 mL of the supernatant was added to 1 mL of 0.1 mM DPPH ethanolic solution. An equal amount of ethanol was used as a control. The mixtures were then stored in the dark at room temperature for 30 min. The absorbance of samples was measured at 517 nm using a UV–Vis spectrophotometer (Ultrospec 2000, Pharmacia Biotech, Cambridge, UK). The DPPH radical activity was calculated using Equation (5):

(5) $$\%\mathrm{DPPH}\;\mathrm{radical}\;\mathrm{scavenging}\;\mathrm{activity}=\frac{\mathrm{Control}\;\mathrm{Absorbance}-\mathrm{Sample}\;\mathrm{Absorbance}}{\mathrm{Control}\;\mathrm{Absorbance}}\times 100$$

The DPPH assay was used as a rapid and widely applied method for evaluating antioxidant activity. The results were interpreted as a comparative indicator among different samples and during storage, rather than as an absolute measure of antioxidant capacity. Complementary assays can provide a more comprehensive evaluation.

The antioxidant stability of the optimized BE-loaded NLC, blank NLC, and pure BE was assessed during one-month storage at 4 °C. The analysis was carried out on days 1, 7, 14, and 28 using the DPPH radical scavenging method described above (Section 2.5.7).

#### 2.5.8. Microbiological Analysis

##### Preparation of Bacterial Suspensions

*Staphylococcus aureus* (ATCC 25923) and *Escherichia coli* (ATCC 25922) were cultured on sterile Mueller–Hinton agar (MHA) plates and incubated at 37 °C for 24 h. Afterwards, a single colony of each strain was inoculated into a sterile tube containing 2 mL of sterile normal saline to adjust the turbidity to the 0.5 McFarland standard. Then, the suspensions were diluted in sterile saline to achieve a final concentration of 3 × 10^5^ CFU/mL.

##### Minimum Inhibitory Concentration (MIC) and Minimum Bactericidal Concentration (MBC)

The broth microdilution method was used to determine the antibacterial activity of free BE, coriander essential oil, BE-loaded NLC, and blank NLC. For MIC determination, 100 µL of Mueller–Hinton Broth (MHB) was poured into the wells of a sterile 96-well microplate. Then, 100 µL of the test samples was added to the wells of the first column and mixed properly. Serial dilution was performed by transferring 100 µL from each well to the next, so that each well received half the concentration of the previous one. Afterward, 100 µL of the prepared bacterial suspension was added to each well, for a total volume of 200 μL. Positive control (bacteria in MHB without sample) and negative control (sample in MHB without bacteria) were included. After incubation at 37 °C for 24 h, the lowest concentration without visible turbidity was recorded as the MIC. For MBC determination, 100 µL from the wells that showed no bacterial growth was subcultured onto MHA plates and incubated for 24 h at 37 °C. The MBC was defined as the lowest concentration of antibacterial agent required to kill 99.9% of the initial bacterial inoculum [22].

### 2.6. Experimental Design and Optimization

In this study, a combined D-optimal mixture design with 19 experimental runs was applied using Design-Expert^®^ software (Version 13.0.5.0, Stat-Ease Inc., Minneapolis, MN, USA) to optimize the NLC formulation. For this purpose, the effects of different surfactant ratios (Tween 80/Tween 20 and PGE/PGPR) on particle size (nm), polydispersity index (PDI), zeta potential, and entrapment efficiency (EE%) were investigated (Table 1).

The formulation variables in Table 1 are expressed as mixture percentages to reflect the experimental design. For reproducibility, the total mass of lipophilic surfactants (PGE + PGPR) was fixed at 250 mg, while the hydrophilic surfactants (Tween 80 + Tween 20) were prepared as a 1% (*w*/*v*) solution in 25 mL of the external aqueous phase. Based on these fixed values, the amount of each component can be directly calculated from the reported percentages.

The optimization criteria were defined as minimizing particle size and PDI, while maximizing the absolute value of zeta potential and entrapment efficiency (EE%). Entrapment stability was not included as a response variable, as stability assessment involves time-dependent measurements over a storage period and is not compatible with the structure of a simultaneous optimization design.

Three-dimensional surface plots with contour lines were used to analyze the effects of the independent variables on the response parameters. To evaluate the adequacy of the fitted models, different statistical parameters were considered, including the coefficient of determination (R^2^), adjusted R^2^, predicted R^2^, lack-of-fit test, and coefficient of variation (CV). In addition, one-way analysis of variance (ANOVA) followed by Duncan’s multiple range test at a significance level of 0.05 was used for statistical comparison of the results using SPSS software (Version 16.0, IBM Corp., Chicago, IL, USA).

## 3. Results and Discussion

Prior to formulation optimisation, the total anthocyanin content of the lyophilized barberry extract was determined by the pH-differential method and found to be 10.2 ± 0.5 mg cyanidin-3-glucoside equivalents per gram of dry extract, consistent with values reported for hydroalcoholic barberry extracts [2,17].

### 3.1. NLC Formulation Optimization

#### 3.1.1. Regression Model Analysis

In the present study, the results of the D-optimal mixture design consisting of 19 experimental runs (Table 1) are presented to evaluate the effects of surfactant ratios on the physicochemical characteristics of nanostructured lipid carriers. A sequential modeling strategy was applied to establish polynomial relationships between formulation variables and responses, and to develop statistically adequate models for prediction and optimization.

Following the experimental design, the collected data were statistically analyzed using Design-Expert software to fit different polynomial models, including linear, quadratic, and cubic models. The adequacy of each model was evaluated through analysis of variance (ANOVA), lack-of-fit tests, and model summary statistics (Table 2 and Table 3). The significance of the models was indicated by high F-values, low *p*-values (*p* < 0.001), and non-significant lack of fit (*p* > 0.05), suggesting that the developed models were suitable for describing the experimental domain (Table 3).

As summarized in Table 2, the quadratic × quadratic model was found to best represent the responses of particle size and zeta potential, while linear × linear and quadratic × linear models were selected for PDI and EE%, respectively. The selected models showed significant regression (*p* < 0.001), with R^2^ values ranging from 0.91 to 0.99. The close agreement between adjusted and predicted R^2^ values (difference < 0.2) indicated that the models were suitable for prediction. Furthermore, the coefficient of variation (CV) for all responses was below 10%, indicating good precision and reproducibility of the experimental data. Adeq Precision values above 4 indicated an adequate signal-to-noise ratio and reliability of the developed models.

Therefore, the selected polynomial models were used for subsequent response surface analysis and optimization. The regression equations describing these relationships are presented as follows:

Y_1_ = 84.62 X_1_X_3_ + 202.05 X_1_X_4_ + 196.71 X_2_X_3_ + 279.74 X_2_X_4_ − 1.11 X_1_X_2_X_3_ − 254.76 X_1_X_2_X_4_ − 170.09 X_1_X_3_X_4_ − 63.57 X_2_X_3_X_4_ + 564.94 X_1_X_2_X_3_X_4_ (6)

Y_2_ = 0.15 X_1_X_3_ + 0.28 X_1_X_4_ + 0.36 X_2_X_3_ + 0.41 X_2_X_4_ (7)

Y_3_ = −21.83 X_1_X_3_ − 19.18 X_1_X_4_ − 21.11 X_2_X_3_ − 17.33 X_2_X_4_ − 16.35 X_1_X_2_X_3_ − 12.16 X_1_X_2_X_4_ − 9.89 X_1_X_3_X_4_ + 4.57 X_2_X_3_X_4_ + 39.93 X_1_X_2_X_3_X_4_ (8)

Y_4_ = 78.24 X_1_X_3_ + 58.00 X_1_X_4_ + 54.16 X_2_X_3_ + 41.86 X_2_X_4_ + 8.19 X_1_X_2_X_3_ + 35.20 X_1_X_2_X_4_ (9)

where Y is the response variable (particle size (Y_1_), PDI (Y_2_), zeta potential (Y_3_), and encapsulation efficiency (Y_4_)), and X represents the independent variables (PGE (X_1_), PGPR (X_2_), Tween 80 (X_3_), and Tween 20 (X_4_)).

#### 3.1.2. Response Surface Analysis

**Figure 1:**
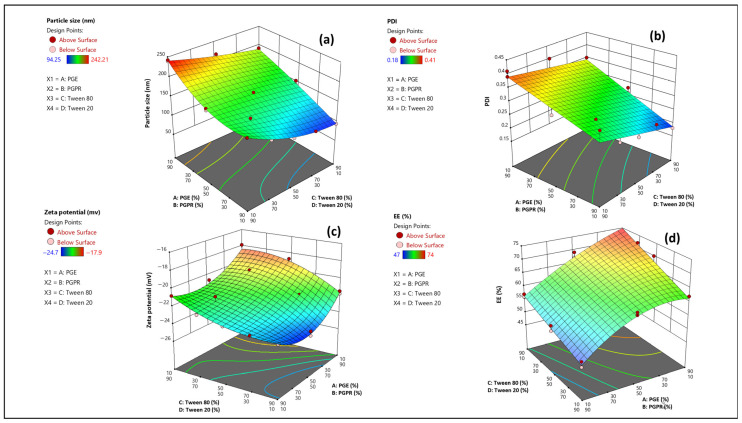
3D response surface plots showing the effects of formulation variables (A: PGE (%), B: PGPR (%), C: Tween 80 (%), and D: Tween 20 (%)) on (**a**) particle size (nm), (**b**) PDI, (**c**) zeta potential (mV), and (**d**) EE (%).

In this study, two mixture variables, Tween 80/Tween 20 ratio and PGE/PGPR ratio, were used to investigate how these surfactant combinations affect particle size, polydispersity index (PDI), zeta potential (ZP), and encapsulation efficiency (EE%) of the NLC formulations (Table 1). Based on the fitted polynomial models obtained from the D-optimal mixture design, three-dimensional response surface and contour plots were generated using Design-Expert software to illustrate the main and interaction effects of the surfactant ratios on each response (Table 2 and Figure 1).

According to the DLS results, the different NLC formulations showed particle sizes ranging from 94.25 to 242.21 nm (Figure 1a). Smaller particles (about 94–112 nm) were obtained in formulations containing a high proportion of PGE (90%) along with higher Tween 80 levels (50–90%), whereas systems with high PGPR contents (70–90%) combined with low Tween 80 levels (10–30%) tended to produce larger particles (>190 nm). For example, at a constant PGE/PGPR ratio of 90/10, increasing Tween 80 from 10 to 90% decreased the mean particle size from 168.13 to 94.25 nm, and at a fixed Tween 80/Tween 20 level of 50/50, increasing PGE from 10 to 90% reduced the size from 215.94 to 112.10 nm. The PDI values were in the range of 0.18 to 0.41 (Figure 1b). The lowest PDI (0.18) was obtained at the highest ratios of PGE/PGPR (90/10) and Tween 80/Tween 20 (90/10), similar to the trend observed for particle size. In colloidal systems, PDI describes the uniformity of particle size distribution; values below 0.3 indicate a narrow and uniform size range, whereas values above 0.5 indicate a broad and non-uniform one.

These results demonstrated that the particle size and PDI were significantly affected by the surfactant type and concentration, in line with previous reports on NLCs [11,19]. In double emulsion systems, a two-step emulsification process usually requires two surfactants, a low-HLB one for stabilizing the primary W_1_/O emulsion and a high-HLB one for stabilizing the external O/W_2_ emulsion [11]. Use of low-molecular-weight surfactants at optimum concentrations can decrease the interfacial tension between the lipid matrix and the aqueous phase and facilitate droplet disruption during homogenization, leading to the formation of smaller NLC particles [22,26]. It has also been reported that lipid-based nanocarriers stabilized with a mixture of surfactants generally exhibit smaller particle size and narrower size distribution than systems prepared with a single surfactant, because appropriate emulsifier combinations improve pre-emulsion stability and limit recrystallization and aggregation of lipid nanocrystals [27].

Another factor that may influence particle characteristics is the effective energy transferred during emulsification. Although the same homogenization and ultrasonication parameters were applied to all runs, changes in surfactant type and ratio can alter the viscosity and density of both phases. As a result, the effective energy density dissipated at the droplet level is not constant across formulations, even under nominally identical processing conditions. In particular, the viscosity ratio (λ = η_d_/η_c_) governs the critical capillary number required for droplet breakup. Accordingly, the lower melt viscosity of PGE-rich formulations likely facilitated more efficient droplet disruption at the W_1_/O interface, partially contributing to the smaller particle sizes observed [28,29].

In the study by Sahraee et al. [14], beeswax-based NLCs stabilized with a Tween 80:PGE ratio of 1:1 showed the smallest mean particle size (≈126 nm) and the lowest PDI (≈0.17) among the tested formulations. This behavior was attributed to the HLB required for beeswax emulsification (around 9–10), given that PGE has an HLB of about 6–8 and Tween 80 of about 14–15, so a 1:1 mixture can provide an effective HLB in this range and allow rapid coverage of the pre-emulsion droplets. Similarly, Khiavi et al. [20] evaluated NLC formulations prepared with Tween 20, Tween 60 or Tween 80 as aqueous-phase surfactants in combination with PGE as a lipid-phase surfactant and reported that Tween 80–based formulations had the smallest mean particle size (103.5 nm) and the lowest PDI (0.18) compared with those containing Tween 20 or Tween 60. In another study, the effects of different polysorbate nonionic surfactants, namely Tween 20, 40, 60, and 80, on NLC were investigated. Formulations containing Tween 80 showed the lowest mean particle size (139.9 ± 15.8 nm), and this size remained in the nano range even after 28 days of incubation, confirming the critical role of the surfactant in stabilizing the NLC. The authors suggested that the bend and kink at the double bond of monooleate in Tween 80 increase the curvature of the NLC particles, which may explain the greater reduction in particle size compared with other Tween types [30].

Zeta potential is commonly measured in emulsion systems to indicate droplet surface charge and the electrostatic repulsion between particles. More negative zeta potential values are associated with stronger repulsive forces and a lower tendency for droplet aggregation and flocculation. In this study, the zeta potential values of the NLC formulations varied from −17.9 to −24.7 mV (Figure 1c). More negative charges (around −25 mV) were observed in samples containing higher levels of Tween 80 combined with intermediate PGE/PGPR ratios (50/50), while formulations with lower Tween 80 contents and higher PGPR levels (70–90%) showed less negative zeta potentials (about −18 to −20 mV). Previous studies have reported that electrostatically stabilized nanosuspensions usually require zeta potential values greater than about ±30 mV for high stability, while sterically stabilized lipid nanoparticles can remain stable at values around ±20 mV due to steric hindrance from the adsorbed nonionic surfactant layer [31]. Since the surfactants used in this study were nonionic, the negative surface charge of the NLCs can be attributed to free fatty acids and carboxylic groups in beeswax, as well as free hydroxyl groups of phenolic compounds present in barberry extract and coriander essential oil. Consistent with these findings, Binazir et al. [25] developed thyme–pennyroyal essential oil-based NLCs loaded with saffron extract using a double emulsion method; the optimal formulation at TEOs/PEOs 90/10 and PGE/PGPR 90/10 showed a zeta potential of −26.7 mV, particle size of 66.87 nm and PDI of 0.38.

Entrapment efficiency in NLC systems depends on several factors, including the type and concentration of surfactants, the ratio and composition of solid and liquid lipids, the solubility of the core material in the NLC matrix, the preparation method, and environmental conditions such as pH and temperature [7]. In addition, the crystalline structure of the nanoparticles may also influence the final %EE. As shown in Figure 1d, the encapsulation efficiency of the formulations ranged from 47 to 74%. The highest EE values (70–74%) were observed in samples containing the highest level of PGE (PGE/PGPR 90/10) together with a high proportion of Tween 80 in the hydrophilic surfactant blend, whereas the lowest EE (47%) occurred in the formulation with the lowest PGE level (PGE/PGPR 10/90) and the lowest Tween 80 content. This behavior can be attributed to the improved interfacial stabilization provided by appropriate surfactant combinations, which reduces the diffusion of hydrophilic compounds into the external aqueous phase and enhances their retention within the internal phase. These findings are consistent with previous reports. Pimentel-Moral et al. [12] used a double-emulsion NLC system to encapsulate *Hibiscus sabdariffa* extract; microwave-assisted extract–loaded NLCs showed EE values of 52.9 ± 0.9% for quercetin and 60 ± 2% for anthocyanins, whereas pressurized liquid extract–loaded NLC showed higher EE values of 93 ± 3% and 84 ± 4%, respectively. In contrast, anthocyanin-loaded W/O/W emulsions formulated with hydrogenated soybean oil (HSO) or soybean oil (SO) and stabilized with PGPR, quillaja saponin and gum arabic showed relatively low initial encapsulation efficiencies (54% for HSO and 44% for SO), which further decreased to about 42% and 33% after 14 days. This reduction was attributed to the expulsion of some anthocyanin-filled droplets during the second homogenization step and to the limited solubility of anthocyanins in the oil phase, which enabled their migration into the external aqueous phase. PGPR was also reported to promote droplet aggregation, consistent with the observed decline in EE over time [32]. Huang and Zhou (2019) reported a microencapsulation efficiency of 99.45 ± 0.24% for anthocyanin-rich black rice extract [33], while Hashemi (2022) obtained an EE of 72% for Pluronic F127 nanoparticles containing barberry extract [34]. An EE of 78.88% was also reported for the optimized NLC formulation loaded with saffron extract [25]. Moreover, in a wax-based pomegranate seed oil NLC, Soleimanian et al. [19] observed EE of 96–99%, which they attributed to the heterogeneous wax–oil structure of the lipid matrix. A similar behavior was reported by Ma et al. [23], who developed beeswax–flaxseed oil NLCs loaded with β-sitosterol using Tween 80 and PGE as surfactants. The optimized formulation achieved nanosized particles (143.9 ± 4.27 nm) with a PDI of 0.229 ± 0.012, a zeta potential of −37.2 ± 1.06 mV, and a high EE of 92.07 ± 2.15%, consistent with the ability of wax–liquid lipid matrices to form less crystalline and more disordered structures that effectively retain encapsulated compounds.

#### 3.1.3. Optimization and Verification of Results

**Figure 2:**
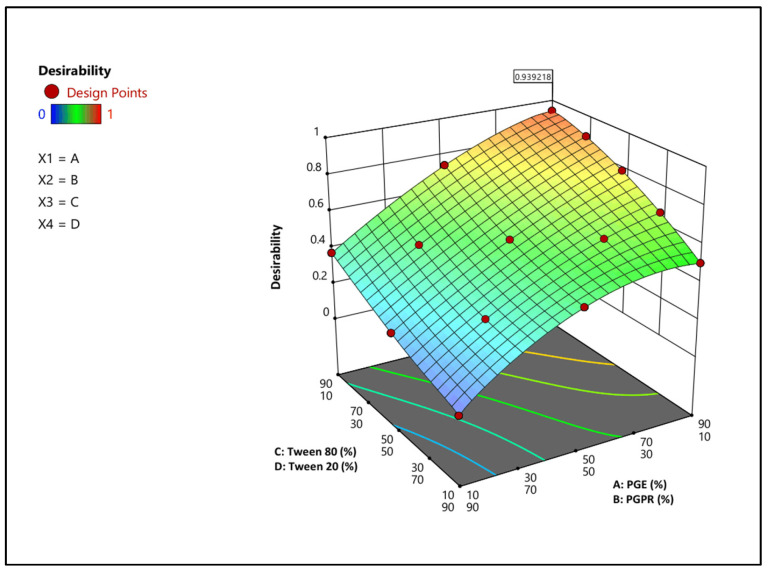
3D desirability surface plot showing the effects of formulation variables (A: PGE (%), B: PGPR (%), C: Tween 80 (%), and D: Tween 20 (%)) on the overall desirability function obtained from numerical optimization.

Optimization of the NLC formulation was performed using the desirability function, with the aim of minimizing particle size and PDI and maximizing the absolute value of zeta potential and entrapment efficiency. The formulation containing PGE/PGPR (90/10) and Tween 80/Tween 20 (90/10) showed the highest desirability value (0.939) and was selected as the optimum formulation. This formulation had a particle size of 94.25 nm, PDI of 0.18, zeta potential of −23.4 mV, and EE of 74%. To assess the validity of the developed models, the experimental values were compared with the corresponding predicted values. The predicted values were in close agreement with the experimental results, indicating the reliability of the developed models for predicting the response variables. The desirability plot and the comparison between predicted and experimental values are presented in Figure 2 and Table 4, respectively.

### 3.2. Entrapment Stability

**Figure 3:**
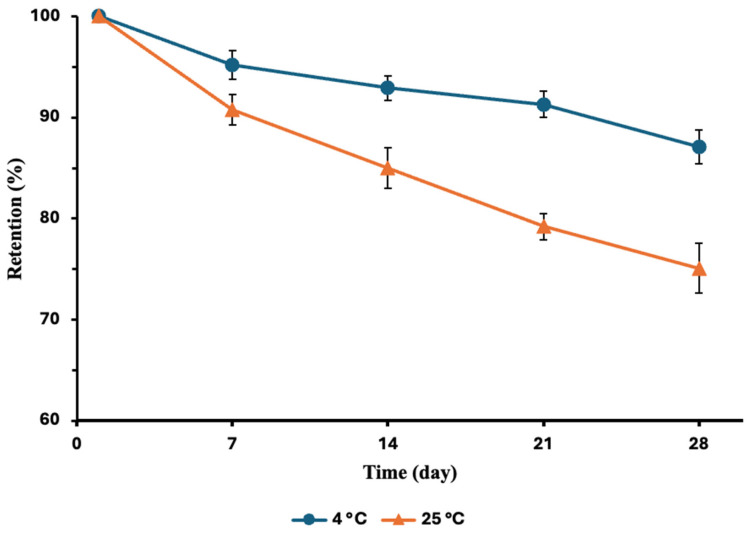
Anthocyanin retention (%) in BE-loaded NLCs during 28 days of storage at 4 °C and 25 °C. Data are presented as mean ± SD (*n* = 3). Retention (%) is expressed relative to day 1 (100%).

The ability of the nanocarriers to retain encapsulated bioactive compounds under different storage conditions is considered an important indicator of their stability. Accordingly, the effect of storage time and temperature (4 °C and 25 °C) on the retention of encapsulated anthocyanins in the optimized NLC formulation during one month is presented in Figure 3. As can be seen, the retention (%) decreased over time, and this decrease was greater at 25 °C than at 4 °C. The lower change observed at 4 °C can be explained by the reduced permeability and flexibility of the lipid matrix and the limited mobility of lipid molecules at this temperature, which slows down oxidative reactions and prevents structural decomposition. Similar results were obtained by Ma et al. [35], who found that the entrapment efficiency of lycopene-loaded NLCs decreased by 16.08% at 25 °C and by 5.95% at 4 °C after 28 days of storage, indicating that refrigerated conditions significantly improve the retention of encapsulated compounds.

### 3.3. Morphology of NLC

**Figure 4:**
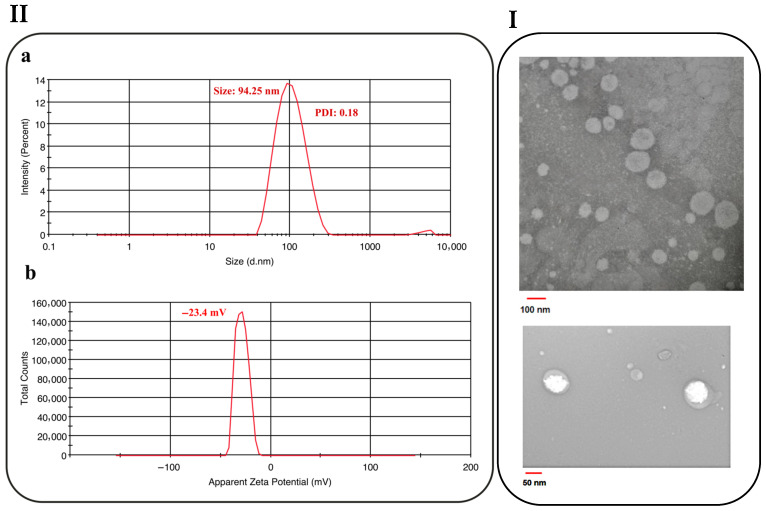
TEM images (**I**), particle size distribution (**IIa**) and zeta potential (**IIb**) of the optimized nanostructured lipid carrier (NLC).

Figure 4I shows the TEM images of the optimized NLC formulation. The micrographs revealed spherical nanoparticles with smooth surfaces. The particles showed a relatively narrow size distribution, and the mean diameter was close to 100 nm, which was consistent with the particle size obtained from the DLS analysis (Figure 4(IIa,IIb)). In some particles, a core–shell-like appearance with electron-density contrast was also observed. The spherical morphology can be regarded as confirmation of a less ordered crystalline structure, whereas elongated particles are related to β modification during lipid crystallization [36]. Similar spherical or ovoid forms with homogeneous distribution and smooth appearance have also been reported for lipid-based nanoparticles in previous studies, supporting the morphological findings of the present formulation [23,27].

### 3.4. FTIR Analysis

**Figure 5:**
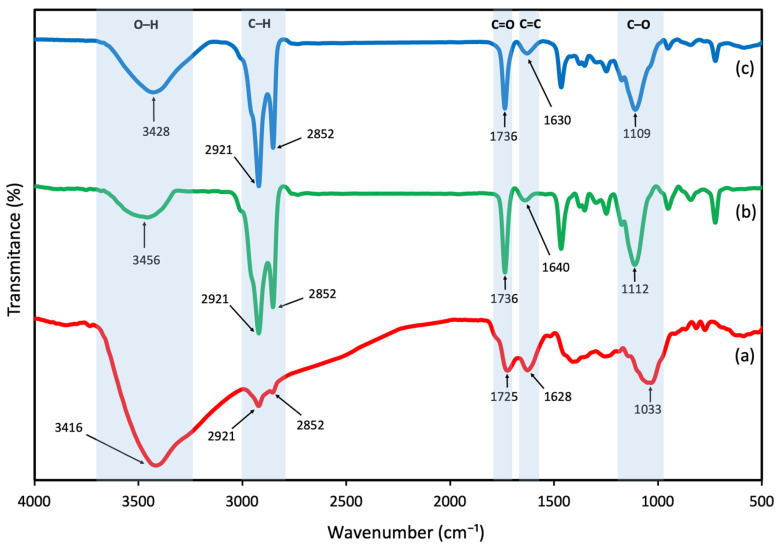
Fourier transform infrared (FTIR) spectra of (**a**) barberry extract (BE), (**b**) blank NLC, and (**c**) barberry extract-loaded NLC (BE-NLC).

FTIR analysis was performed as a qualitative method to identify functional groups, verify the presence of bioactive compounds in the lipid matrix, and detect potential interactions between components. The FTIR spectra of barberry extract, blank NLC, and extract-loaded NLC were recorded in the range of 400–4000 cm^−1^ and are shown in Figure 5. The FTIR spectrum of the barberry extract showed a characteristic peak at 3416 cm^−1^, attributed to the stretching vibrations of hydroxyl groups (OH) of phenols and sugars. The bands observed at 2921 and 2852 cm^−1^ corresponded to the asymmetric and symmetric C–H stretching vibrations of aliphatic –CH_2_ and –CH_3_ groups, respectively. A distinct peak at 1725 cm^−1^ was assigned to the C=O stretching vibration, which is characteristic of carbonyl groups of carboxylic acids and phenolic esters. The band at 1628 cm^−1^ represented aromatic C=C stretching vibrations, typical of the flavonoid backbone in anthocyanin compounds. Another band observed at 1408 cm^−1^ was related to C–C stretching within the aromatic ring. Additionally, the band at 1033 cm^−1^ was attributed to C-O stretching vibrations, indicating the presence of carbohydrate moieties in the glycosylated anthocyanins which was also confirmed by Homayoonfal et al. [4]. In blank NLC, a broad band at 3456 cm^−1^ corresponded to O–H stretching vibrations of hydroxyl groups in surfactants and residual bound moisture. Strong absorption bands at 2921 cm^−1^ and 2852 cm^−1^ were related to the asymmetric and symmetric C–H stretching vibrations of aliphatic –CH_2_ groups in beeswax and essential oil components. The band at 1736 cm^−1^ was assigned to the C=O stretching vibration of ester linkages, particularly from wax esters in beeswax [37]. A band at 1640 cm^−1^ was attributed to aromatic C=C stretching, related to phenolic compounds present in coriander essential oil [12,25]. Methylene bending vibrations were observed at 1466 cm^−1^, typical of CH_2_ scissoring in long-chain fatty acids and wax components [27]. The band at 1112 cm^−1^ was associated with C–O stretching vibrations, in agreement with previous studies that reported similar peaks for surfactant molecules and ester linkages in NLC formulations [21,38]. By comparing the spectra of blank and extract-loaded NLC, differences in band positions and intensities were observed, providing evidence for incorporation of the extract into the lipid matrix. These changes were mainly found in the O–H and aromatic C=C regions. The O–H stretching band shifted from 3456 cm^−1^ in blank NLC to 3428 cm^−1^ in the loaded formulation, representing a shift of 28 cm^−1^ toward lower frequency. This shift indicates the formation of hydrogen bonding interactions between the phenolic hydroxyl groups of the barberry extract and the lipid matrix components. The aromatic C=C stretching band shifted from 1640 cm^−1^ to 1630 cm^−1^, indicating interactions between aromatic components. The presence of this characteristic band in the spectrum of extract-loaded NLC confirmed the effective encapsulation of the extract within the system, consistent with previous reports for other phenolic extracts [12,20]. The preservation of major functional group bands with slight frequency shifts suggested that encapsulation occurred through non-covalent interactions rather than chemical bond formation.

### 3.5. Thermal Analysis

**Figure 6:**
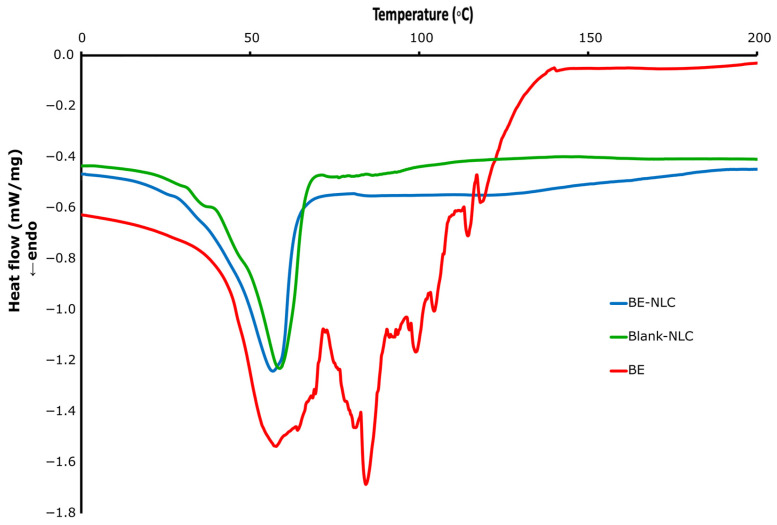
Differential scanning calorimetry (DSC) of barberry extract (BE), blank NLC, and barberry extract-loaded NLC (BE-NLC).

Differential scanning calorimetry (DSC) is used to analyze the melting and crystallization characteristics of nanocarriers and to identify thermal transitions related to lipid organization. This technique determines variations in temperature and enthalpy at phase transitions to describe the crystalline arrangement of the matrix. Shifts in peak position or intensity indicate changes in lattice order and crystal packing, confirming the interaction between the lipid matrix and the encapsulated bioactive material [8]. The DSC thermograms of BE, blank NLC, and extract-loaded NLC are shown in Figure 6. The barberry extract exhibited two sharp endothermic peaks at 131.7 °C and 154.9 °C, indicating its crystalline nature and polymorphic transitions. The blank NLC presented a distinct endothermic melting peak at 57.8 °C, corresponding to the melting of the lipid phase. The heat flow profile of the extract-loaded NLC was similar to that of the blank NLC, with a slight shift in the melting peak toward a lower temperature (56.5 °C). The decrease in melting temperature can be attributed to the interaction between the extract and lipid components, leading to a decrease in lattice order [7]. The disappearance of the extract’s characteristic peaks in the thermogram of the loaded NLC suggested uniform dispersion of the extract within the lipid matrix and loss of its crystalline form. These findings were in accordance with previous studies [21,22]. Vardanega et al. [39] reported that incorporation of cannabidiol extracts (CBDiso and CBDext) into NLCs reduced both the melting temperature and enthalpy compared to unloaded systems, indicating the formation of amorphous regions and a decrease in lipid crystallinity. Liu et al. [24] investigated curcumin-loaded nanostructured lipid carriers composed of stearic acid and conjugated linoleic acid to evaluate the effect of bioactive incorporation on lipid crystallinity. The melting peak of solid stearic acid appeared at 75.7 °C, while that of the mixed lipids shifted to 66.5 °C and further decreased to 55.6 °C after curcumin loading. This reduction in melting temperature and the absence of curcumin’s endothermic peak indicated the formation of a less ordered lipid matrix and the successful incorporation of the compound into the carrier system.

### 3.6. XRD Analysis

**Figure 7:**
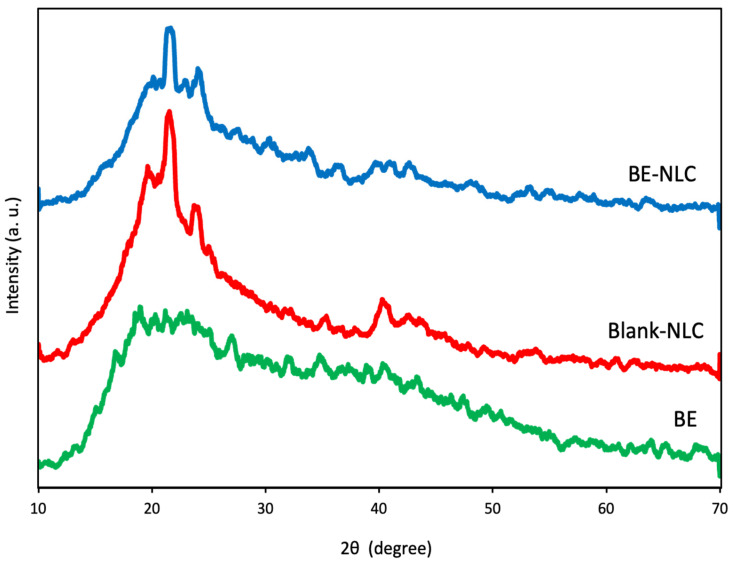
X-ray diffraction (XRD) patterns of barberry extract (BE), blank NLC, and barberry extract-loaded NLC (BE-NLC).

XRD is used to determine the crystal forms and degree of crystallinity in lipid carriers and to detect polymorphic transitions after preparation [8]. It also provides information on the compatibility between lipid carriers and encapsulated compounds, which influences nanoparticle stability and release properties [7]. Figure 7 shows the diffractograms of BE, blank NLC, and extract-loaded NLC. The crystalline nature of barberry extract was demonstrated by significant peaks at 2θ = 18.78°, 20.52°, and 20.90° with d-spacings of 4.721, 4.325, and 4.247 Å, respectively. These peaks indicate the presence of β and β′ polymorphic forms, confirming the crystalline structure of the extract, which could be attributed to the presence of polyphenolic compounds, salts, and sugars.

In the blank NLC, a dominant diffraction peak was observed at 2θ = 21.58° (d = 4.115 Å), corresponding to the α polymorphic form. Two medium intensity reflections appeared at 19.26° (d = 4.605 Å) and 23.86° (d = 3.726 Å), which are attributed to β and β′ crystals, respectively. These reflections reveal the characteristic crystalline phases of the beeswax-based lipid matrix, in agreement with previous reports on wax-containing NLC systems [14,19]. In addition, a weak diffraction feature was observed at 2θ = 40.48° (d = 2.23 Å), which can be associated with the molecular packing of long-chain hydrocarbon components of beeswax.

The XRD pattern of extract-loaded NLC showed an α reflection at 2θ = 21.60° (d = 4.111 Å), with β′ peaks at 20.46° (d = 4.337 Å) and 23.92° (d = 3.717 Å). A weak β-like signal near 19.62° (d = 4.521 Å) was also noted. Compared with the blank NLC, the peak intensities were lower and slight shifts in 2θ were observed, indicating a less ordered crystalline structure in the loaded system. Polymorphic modifications in the lipid matrix affect the crystalline arrangement and the ability of the system to incorporate and retain bioactive compounds. The presence of a less ordered crystalline lipid matrix in NLC creates more imperfections and voids, leading to higher encapsulation efficiency, minimized expulsion of encapsulant over time, and enabled controlled release. Internal factors, including lipid type, impurity and surfactant nature, as well as external parameters such as temperature, pressure, and cooling rate, affect the crystalline state of the lipid matrix and the stability of nanoparticles during storage [7]. Nasr et al. [27] investigated beeswax-based NLCs loaded with naringenin and ferulic acid. Their XRD analysis showed that the incorporation of these polyphenols into the carrier resulted in the disappearance of sharp crystalline peaks and a decrease in crystallinity, indicating successful loading into the lipid matrix. In contrast to the present findings, Malekmohammadi et al. [22] reported that loading sage extract into NLCs resulted in an increase in peak intensity in their XRD patterns, indicating higher crystallinity compared to the unloaded carriers. The blank NLCs displayed characteristic peaks related to intermediate β–β′ and sub-α polymorphs, while extract incorporation was associated with the formation of more ordered crystalline structures.

### 3.7. Antioxidant Activity

**Figure 8:**
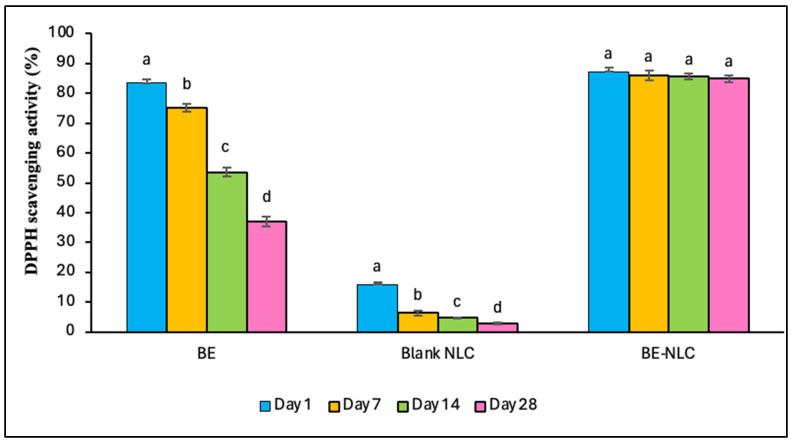
DPPH scavenging activity of barberry extract (BE) (1000 μg BE/mL), blank NLC, and BE-NLC (containing 1000 μg BE/mL) during one month. Different letters in each group demonstrate significant differences at the 5% level in Duncan’s test.

Antioxidants are chemical substances that prevent oxidative stress, inflammation, and cellular damage by scavenging reactive oxygen species through donating hydrogen atoms or electrons [40]. In this experiment, the antioxidant activity of the free BE, blank NLC, and BE-loaded NLC was determined using the DPPH assay during one-month storage at 4 °C (Figure 8). The day after preparation, the antioxidant activity of the free and encapsulated barberry extract at the concentration of 1000 μg extract/mL was 83.4 ± 1.12% and 87.25 ± 1.35%, respectively. The antioxidant activity of the blank NLC was 16.0 ± 0.82%, which may be related to the composition of the carrier materials. The presence of *Coriandrum sativum* essential oil as the liquid lipid phase can provide inherent antioxidant activity due to its terpenoid constituents, mainly linalool, which possesses hydrogen-donating ability and can react with free radicals [41]. Beeswax, as the solid lipid phase, contains long-chain fatty acids and minor phenolic residues that slightly contribute to radical scavenging [42]. Nonionic surfactants (Tween 20 and Tween 80) may also reduce oxidative propagation at the lipid–aqueous interface by stabilizing the system [43]. After 28 days of storage, the antioxidant capacity decreased in all samples. However, this decrement was not significant (*p* > 0.05) for the encapsulated extract, indicating that its antioxidant properties remained stable over one month of storage. Although a decrease in anthocyanin content was observed during storage, the DPPH radical-scavenging activity of the BE-loaded NLC remained relatively unchanged, which may be attributed to the contribution of other components in the system, including the essential oil and phenolic compounds. This stability could also be related to the protective effect of the lipid matrix of the NLC system against environmental conditions. Similar findings have been reported in previous studies, where encapsulation prevented the decrease in antioxidant activity over time [22,25].

On the other hand, the free extract showed a reduction of about 55%, which may be attributed to the oxidation and degradation of phenolic compounds in the presence of oxygen. The antioxidant activity of barberry extract is directly correlated with its anthocyanin and phenolic compounds, which are responsible for its radical-scavenging capacity [2]. This relationship has also been demonstrated by Jaberi et al. [17], who reported that under optimum extraction conditions (80% ethanol and 2% citric acid), barberry extract showed a total phenolic content of 3269.05 ± 111.11 mg GAE kg^−1^ and a DPPH radical-scavenging activity of 92.41 ± 0.25%, confirming its high antioxidant capacity.

### 3.8. Antimicrobial Activity

The MIC and MBC values of barberry extract (BE), coriander essential oil (CEO), blank NLC, and extract-loaded NLC against *S. aureus* and *E. coli* are shown in Table 5. According to the obtained results, the lowest MIC and MBC values were observed for coriander essential oil (0.23 and 0.47 mg/mL for *S. aureus*, and 0.47 and 0.94 mg/mL for *E. coli*), indicating the strongest antibacterial activity among all tested samples. This effect is attributed to the presence of major volatile compounds such as linalool, α-pinene, p-cymene, γ-terpinene, limonene, and linalyl acetate, which can interact with the phospholipid bilayer of bacterial membranes, increasing permeability and causing leakage of cellular contents [15]. *S. aureus* was more sensitive than *E. coli*, which can be associated with differences in their cell wall structures. Gram-negative bacteria possess an outer membrane rich in lipopolysaccharides that limits the penetration of hydrophobic compounds, while Gram-positive bacteria contain a thick peptidoglycan layer with lipoteichoic acids that facilitate adsorption and diffusion of essential oil constituents [41,44]. As can be seen in Table 5, barberry extract required higher concentrations to inhibit bacterial growth and was also more effective against *S. aureus* (MIC = 3.75 mg/mL, MBC = 7.5 mg/mL) than *E. coli* (MIC = MBC = 15 mg/mL). These values are within the ranges reported by Mojaddar Langroodi et al. [45] for ethanolic (3.12–12.5 mg/mL) and aqueous (6.25–25 mg/mL) barberry extracts. Variations in MIC and MBC values among different studies may be related to the solvent system, extraction conditions, and the type of bacterial strain tested [46]. The antibacterial properties of barberry extract have been mainly attributed to its polyphenolic compounds, which can interfere with bacterial membranes and inhibit cell growth [34]. The extract-loaded NLC showed stronger antibacterial activity compared with the free extract, which could be related to the presence of coriander essential oil in the lipid matrix [15]. The blank NLC also exhibited similar effects to the extract-loaded NLC, suggesting that the incorporation of barberry extract had no considerable impact on the initial antibacterial activity.

## 4. Conclusions

In this study, anthocyanin-rich barberry extract was successfully entrapped in a nanostructured lipid carrier system prepared by the water-in-oil-in-water double emulsion technique. Optimization of surfactant ratios resulted in a formulation with high encapsulation efficiency, small particle size, narrow polydispersity index, and a negative zeta potential. Infrared and X-ray diffraction analyses supported interactions between the extract and carrier components, consistent with successful incorporation into the lipid matrix. Storage studies demonstrated that the antioxidant activity of anthocyanin-rich barberry extract was better preserved after encapsulation compared with the free extract. In addition, the extract-loaded nanostructured lipid carrier exhibited stronger antibacterial activity against *S. aureus* and *E. coli* than the free extract mainly due to the presence of coriander essential oil in its formulation. These findings indicate that the developed food-grade nanostructured lipid carrier system can be considered a suitable carrier for improving the stability and functionality of anthocyanin-rich barberry extract in food applications.

## Figures and Tables

**Table 1:** Combined D-optimal mixture design and corresponding responses of NLC formulations.

Runs	Components		Components		Responses			
	Mixture Components 1		Mixture Components 2		Particle Size (nm)	PDI	ZP (mV)	EE (%)
	X1: PGE (%) ^a^	X2: PGPR (%) ^b^	X3: Tween 80 (%) ^c^	X4: Tween 20 (%) ^d^				
1	10	90	90	10	191.53	0.33	−21.5	57
2	30	70	30	70	192.16	0.34	−19.8	55
3	10	90	10	90	239.32	0.41	−18.9	47
4	50	50	90	10	152.92	0.26	−24.2	67
5	30	70	70	30	171.4	0.3	−21.6	59
6	50	50	50	50	168.45	0.29	−22	63
7	90	10	30	70	134.6	0.24	−22.3	62
8	50	50	10	90	177.29	0.31	−21.6	61
9	90	10	70	30	101.55	0.23	−23.2	72
10	70	30	30	70	157.22	0.29	−21.2	64
11	90	10	90	10	94.25	0.18	−23.4	74
12	50	50	10	90	172.8	0.31	−20.7	60
13	10	90	90	10	188.71	0.35	−21.8	56
14	10	90	50	50	210.7	0.4	−18.7	53
15	90	10	50	50	112.1	0.22	−22.9	70
16	50	50	90	10	142.14	0.28	−24.7	69
17	10	90	50	50	215.94	0.36	−19.2	51
18	10	90	10	90	242.21	0.39	−17.9	49
19	90	10	10	90	168.13	0.32	−20.8	62

^a^ This value indicates the ratio of PGE to the total PGE + PGPR. ^b^ This value indicates the ratio of PGPR to the total PGE + PGPR. ^c^ This value indicates the ratio of Tween 80 to the total Tween 80 + Tween 20. ^d^ This value indicates the ratio of Tween 20 to the total Tween 80 + Tween 20.

**Table 2:** Fitted models and summary statistics for the response variables in the combined D-optimal design.

Source	Suggested Models		Sequential *p*-Value		Fit Statistics			CV (%)	Adeq. Precision
	Mix Order 1	Mix Order 2	Mix 1	Mix 2	R^2^	Adj. R^2^	Pred. R^2^		
Particle size	Quadratic	Quadratic	0.0006 ***	0.0375 *	0.9924	0.9863	0.9775	2.88	43.1751
PDI	Linear	Linear	<0.0001 ***	0.0002 ***	0.9104	0.8925	0.8296	6.70	21.7278
ZP	Quadratic	Quadratic	0.0005 ***	0.0068 **	0.9722	0.9499	0.9018	1.97	20.9380
EE	Quadratic	Linear	0.0008 **	<0.0001 ***	0.9742	0.9643	0.9448	2.40	33.2168

*, **, and *** indicate statistical significance at *p* < 0.05, *p* < 0.01, and *p* < 0.001, respectively.

**Table 3:** Regression coefficients for the response variables and analysis of variance of the regression models.

Source	Particle Size		PDI		ZP		EE	
	F-Value	*p*-Value	F-Value	*p*-Value	F-Value	*p*-Value	F-Value	*p*-Value
Model	162.54	<0.0001 ***	50.82	<0.0001 ***	43.67	<0.0001 ***	98.32	<0.0001 ***
Linear x Linear Mixture	417.78	<0.0001 ***	50.82	<0.0001 ***	96.40	<0.0001 ***	155.16	<0.0001 ***
ABC	0.7627	0.4030	—	—	26.44	0.0004 ***	2.16	0.1652
ABD	41.19	<0.0001 ***	—	—	14.11	0.0037 **	19.89	0.0006 ***
ACD	11.14	0.0075 **	—	—	3.01	0.1134	—	—
BCD	0.7996	0.3922	—	—	8.93	0.0136 *	—	—
ABCD	6.29	0.0310 *	—	—	4.25	0.0663	—	—
Lack of Fit	1.66	0.2953	1.75	0.2794	0.4777	0.7816	1.83	0.2614

*, **, and *** indicate statistical significance at *p* < 0.05, *p* < 0.01, and *p* < 0.001, respectively. Non-significant terms were retained to preserve the hierarchical structure of the model.

**Table 4:** Optimum formulation and corresponding predicted and experimental responses.

Optimum Formulation				
PGE (%)	PGPR (%)	Tween 80 (%)	Tween 20 (%)	Desirability
90	10	90	10	0.939
Responses at the optimum point		Predicted	Experimental	Percentage error
Particle size (nm)		95.07	94.25	+0.87
PDI		0.186	0.180	+3.33
Zeta potential (mV)		−23.35	−23.40	−0.21
EE (%)		74.87	74.00	+1.18

**Table 5:** MIC and MBC results of barberry extract (BE), coriander essential oil (CEO), blank NLC, and barberry extract-loaded NLC (BE-NLC) against *S. aureus* and *E. coli*.

	Bacteria	*S. aureus*		*E. coli*	
Sample					
		MIC (mg/mL)	MBC (mg/mL)	MIC (mg/mL)	MBC (mg/mL)
BE		3.75	7.5	15	15
CEO		0.23	0.47	0.47	0.94
Blank NLC		0.47	0.94	0.94	1.88
BE-NLC		0.47	0.94	0.94	1.88

## Data Availability

The original contributions presented in this study are included in the article. Further inquiries can be directed to the corresponding author.
